# Neuroprotection: Targeting Multiple Pathways by Naturally Occurring Phytochemicals

**DOI:** 10.3390/biomedicines8080284

**Published:** 2020-08-12

**Authors:** Andleeb Khan, Sadaf Jahan, Zuha Imtiyaz, Saeed Alshahrani, Hafiz Antar Makeen, Bader Mohammed Alshehri, Ajay Kumar, Azher Arafah, Muneeb U. Rehman

**Affiliations:** 1 Department of Pharmacology and Toxicology, College of Pharmacy, Jazan University, Jazan 45142, Saudi Arabia; salshahranipharma@gmail.com; 2 Medical Laboratories Department, College of Applied Medical Sciences, Majmaah University, Majmaah 15341, Saudi Arabia; jahan149@gmail.com (S.J.); b.alshehri@mu.edu.sa (B.M.A.); 3 Clinical Drug Development, College of Pharmacy, Taipei Medical University, Taipei 11031, Taiwan; zuhabazaz161991@gmail.com; 4 Department of Clinical Pharmacy, College of Pharmacy, Jazan University, Jazan 45142, Saudi Arabia; hafizamakeen1@gmail.com; 5 Institute of Nano Science and Technology, Habitat Centre, Phase-10, Sector-64, Mohali 160062, India; ajaypharm.11@gmail.com; 6 Department of Clinical Pharmacy, College of Pharmacy, King Saud University, Riyadh 11451, Saudi Arabia; aazher@ksu.edu.sa (A.A.); muneebjh@gmail.com (M.U.R.)

**Keywords:** neurodegenerative diseases, phytochemicals, natural products, neuroprotection

## Abstract

With the increase in the expectancy of the life span of humans, neurodegenerative diseases (NDs) have imposed a considerable burden on the family, society, and nation. In defiance of the breakthroughs in the knowledge of the pathogenesis and underlying mechanisms of various NDs, very little success has been achieved in developing effective therapies. This review draws a bead on the availability of the nutraceuticals to date for various NDs (Alzheimer’s disease, Parkinson’s disease, Amyotrophic lateral sclerosis, Huntington’s disease, vascular cognitive impairment, Prion disease, Spinocerebellar ataxia, Spinal muscular atrophy, Frontotemporal dementia, and Pick’s disease) focusing on their various mechanisms of action in various in vivo and in vitro models of NDs. This review is distinctive in its compilation to critically review preclinical and clinical studies of the maximum phytochemicals in amelioration and prevention of almost all kinds of neurodegenerative diseases and address their possible mechanism of action. PubMed, Embase, and Cochrane Library searches were used for preclinical studies, while ClinicalTrials.gov and PubMed were searched for clinical updates. The results from preclinical studies demonstrate the efficacious effects of the phytochemicals in various NDs while clinical reports showing mixed results with promise for phytochemical use as an adjunct to the conventional treatment in various NDs. These studies together suggest that phytochemicals can significantly act upon different mechanisms of disease such as oxidative stress, inflammation, apoptotic pathways, and gene regulation. However, further clinical studies are needed that should include the appropriate biomarkers of NDs and the effect of phytochemicals on them as well as targeting the appropriate population.

## 1. Introduction

Various conditions affecting nerve cells and the nervous system due to the loss of neurons and their connecting networks are described under the superordinate phrase “Neurodegenerative diseases”. They lead to disability due to gradual neuronal death in both the central nervous system (CNS) and the peripheral nervous system (PNS). Several diseases are genetic with few coming being caused due to the exposure to various toxins and chemicals. The main symptoms associated with these disorders are related to movements (ataxia) or mental functioning (dementia) or both causing morbidity and death. This fostered the profound social and economic implications [1]. The most common NDs include cognitive and behavioral disorders, Alzheimer’s disease (AD), Parkinson’s disease (PD), Amyotrophic lateral sclerosis (ALS), Huntington’s disease (HD), Spinocerebellar ataxia (SCA), Spinal muscular atrophy (SMA), Vascular cognitive impairment Prion disease, Frontotemporal disease, Pick’s disease, etc. The treatment available for these disorders gives only symptomatic relief to the patient by extending the lifespan to a few years [2]. Still, a lot of research is in progress to find the therapeutic markers in these diseases [3].

There has been an extensive approach to non-pharmacological therapies to combat the ill effects of NDs. Many of them include disease-modifying therapies, non-invasive brain stimulation techniques, physical exercise, adaptive physical activity, complementary and alternative medicine, and several nutraceutical compounds. In a meta-analysis, it was reported that repetitive transcranial magnetic stimulation (rTMS) therapy with Parkinson’s patients resulted in mild to moderate improvement of motor activities [4]. In another study, it was reported that both rTMS and transcranial direct current stimulation (tDCS) have shown improvement in the cognitive performance of Alzheimer’s patients [5]. Fisicaro and colleagues stated in their review that rTMS can be effectively used as a non-pharmacological tool for various motor and non-motor neurorehabilitation. They suggested that this technique along with other conventional rehabilitative modalities can be effectively used in clinics but still the regime is not clear [6]. Physical exercise has been a promising therapy for motor deficits for years. It could have therapeutic potential for the prevention of mental disorders and neurodegeneration. Extensive research to explain different molecular pathways involved in this therapy is under progress, suggesting multiple pathways may be involved together in neuroprotection [7,8]. The major cornerstones in managing ataxic patients are physiotherapy and kinesitherapy in the present scenario. Studies confirm that customized programs for various exercises of coordination, balance, cognitive skills, and posture maintenance have shown extensive improvement along with the conventional pharmacological therapeutics [9]. Complementary and alternative medicines have always attracted various disease prevention including NDs [10,11]. In addition to these, the various nutraceuticals [12] and phytochemicals multi-target approaches have proved to be a promising adjunct to the present treatment [13].

**Figure 1:**
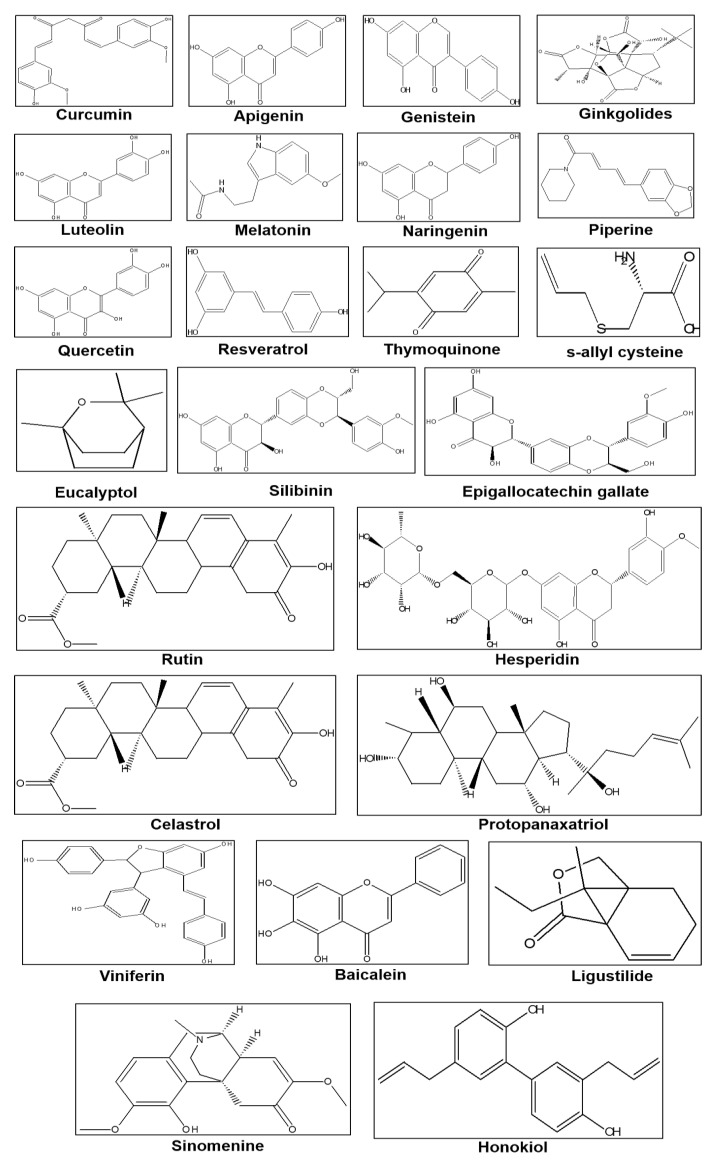
Chemical structures of some common phytochemicals used in the prevention of neurodegenerative diseases (NDs).

The large number of pharmacological or biological activities of the phytochemicals have made them appropriate candidates for the treatment of the NDs [14]. They exhibit antioxidant (resveratrol, zingerone), anti-inflammatory property (cineole, thymoquinone), inhibitors of gamma-aminobutyric acid (GABA_A_) receptors (diterpenes and cyclodepsipeptides), and Monoamine oxidase-B (MAO-B) receptors (selegiline rasagiline), although not proven clinically. Combating disease symptoms by phytochemicals and herbal nutraceutical is known in traditional medicines, and several new research for preventing NDs by phytochemicals are under progress [15]. In this review, our focal point is to discuss the recent advancements in the field of neuroprotection related to the NDs by various phytochemicals and to elicit their potential mechanism of actions. In particular, we discuss phytochemicals used in the prevention of all kinds of NDs and related signaling pathways and mechanism of recovery highlighting the role of phytochemicals. The basic structures of most the common neuroprotective phytochemicals are given in Figure 1.

## 2. Methodology

A Medline (PubMed), Cochrane Library and Embase based literature survey was performed by using keywords of “neurodegenerative diseases, prevention, non-pharmacological therapies, and neurodegenerative diseases, phytochemicals, Alzheimer’s disease natural products, Parkinson’s disease (PD) phytochemicals, Huntington’s disease phytoprotection, natural product amyotrophic lateral sclerosis, phytochemicals cerebral ischemia, phytochemicals prion disease, spinocerebellar ataxia prevention by natural products, natural product and spinal muscular atrophy, frontotemporal dementia natural products, pick’s disease, and natural products” till June 2020.

Two independent authors (A.K. and S.J.) screened all the titles and abstracts of the retrieved data, disagreements were resolved by the consensus of a third author (Z.I.). Duplicated entries, retracted publications, studies on other diseases or conditions different from NDs or its subtypes, studies without statistical analysis, non-English written papers, publications that are not research studies (i.e., commentaries, letters, editorials, reviews, meta-analysis), and any other article that did not fit within the scope of this review, were excluded. Articles listed in the references were also reviewed in search of more data.

**Figure 2:**
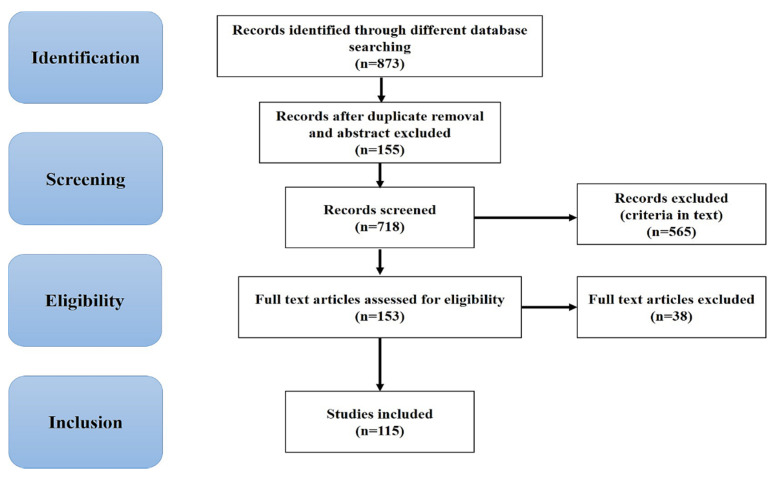
Flow diagram showing the search strategy, the number of records identified, and the included/excluded studies [16].

A total of 873 results were retrieved and screened with the above keywords. Out of these 115 publications were selected and eventually used for qualitative analysis (Figure 2) and the same are summarized in Table 1. In more detail, 28 studies work with AD, 17 with PD, 7 with ALS, 8 with HD, 20 with VCI and stroke, 4 with prion, 7 with FTD, 2 with SMA, and 1 with SCA and others on non-pharmacological therapy and neurodegenerative diseases.

## 3. Multifunctional Phytochemicals as Novel Therapeutic Agents for Neurodegenerative Disorders

### 3.1. Alzheimer’s Disease and Related Dementias

**Figure 3:**
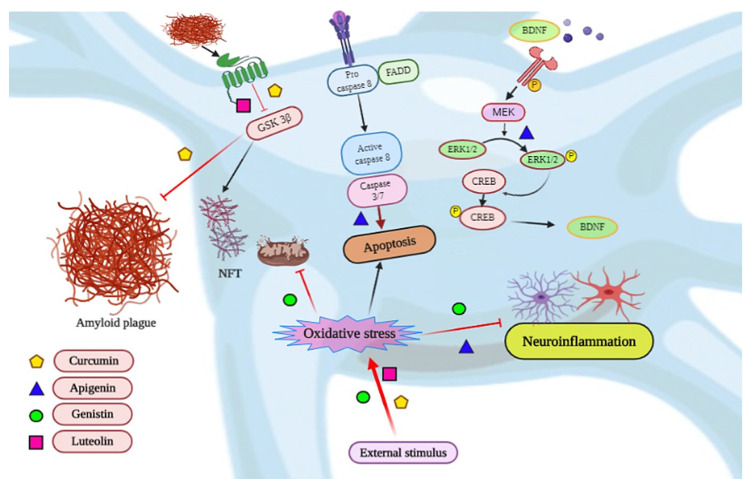
Schematic representation of the several mechanisms associated with Alzheimer’s Disease (AD) and their possible prevention by some phytochemicals. The main hallmarks of AD; amyloid plague and NFT are prevented by curcumin and luteolin via GSK 3β pathway. Curcumin, genistin, and luteolin decreases the oxidative stress in the neuron. Neuroinflammation is also inhibited by genistin and apigenin. The pathway of apoptosis, activated in AD is blocked by the use of apigenin. Apigenin also activates BDNF/ERK/CREB pathway leading to neuronal survival.

AD is a multifactorial, pernicious disease with genetic and environmental factors leading to irreversible cognitive impairment [17]. The main neuropathological features of AD comprises of neuronal loss, amyloid-β plaques, and neurofibrillary tangles in limbic and neocortical regions [18]. Various mechanisms are associated with the progression of the disorder which includes amyloid deposition, tau hyperphosphorylation and aggregation, loss of cholinergic system, oxidative stress, inflammation, apoptosis, glutamatergic excitotoxicity, and decrease in neurogenesis and neurotrophic factors [17,19,20]. In recent years, approved drugs for AD are only modulators of cholinergic and glutamatergic systems, which can only delay the symptoms to some extent [21]. Divergent antioxidant and anti-inflammatory medications are also approved for AD patients [22]. There are recent reports that herbal products and nutraceuticals can act as a multi-target approach as suggested in the traditional system of medicines [23]. Recently extensive research has been conducted which supports their potential in in vitro, in vivo, and clinical studies. In the list of natural compounds, curcumin is considered on the top because of its miraculous qualities. In this section, the mechanistic approach of the curcumin and other important natural compounds will be discussed. Curcumin is a polyphenolic compound and a natural herb which is an integrative part of every Indian kitchen. In literature, many beneficial properties are reported like anti-inflammatory, anti-cancerous, anti-apoptotic, antioxidative, neuroprotective [24]. Apart from that curcumin is also reported as a therapeutic agent to treat rheumatoid disorders, cough, and neurological disorders. Fortunately, it has intensive effects and have multiple sites of action [25]. The relationship between curcumin consumption and a lower prevalence of AD has been observed. In one study, curcumin emulates the improvement of the spatial learning and memory in the rat model and it is reported as an inhibitor of BAC1 in in vitro study. Curcumin has a promising effect in neuroblastoma SHSY5Y cell lines and also has a therapeutic effect on the AD-induced mechanisms [26]. Acrolein exposed HT22 murine hippocampal neuronal cells were protected by curcumin by following the BDNF/TrkB signaling [27]. Orally administrated curcumin downregulates the level of GSK3β and also inhibit the hippocampus Aβ plaques with the improved cognitive impairment and improved maze test in in vivo study [28]. Curcumin effectively counteracted the p25-mediated glial activation and pro-inflammatory chemokines/cytokines production in p25Tg mice. Moreover, this curcumin-mediated suppression of neuro-inflammation reduced the progression of p25-induced tau/amyloid pathology and in turn ameliorated the p25-induced cognitive impairments [29]. Therefore, through the series of research, curcumin is found to be promising phytochemicals against Aβ accumulation and would be a determined remedy against AD. Pilot study with curcumin and *Ginkgo* on 34 possible or probable AD patients was carried out with curcumin and *Ginkgo* extract. It was found that no side effects of curcumin along with that mini-mental examination scores or plasma amyloid beta40 level between 0 and 6 months [30]. AD-related pathology in the brain is well documented, the disease has also been reported to affect the retina, a developmental outgrowth of the brain, which is more accessible for imaging. Early detection of AD, utilizing curcumin fluorescence imaging to highlight Aβ aggregates in the retina. Results show that retinal fluorescence imaging is justified with brain plaque burden from PET scan [31]. Apigenin, a known phytochemical also targets the AD by its antioxidative effects. In plants, Apigenin is found as aglycon and more often in glycoside form. Apigenin inhibits the copper-mediated *β*-amyloid neurotoxicity (copper-induced) via antioxidative mechanism followed by MAPK signal inactivation in an AD cell model [32]. The Apigenin follows the ERK/CREB/BDNF pathway for neuroprotection [33]. On the list of phytochemical-induced mechanistic pathways, genistin also has an important role in neuroprotection. It is a component of the soya bean meal. The effect of genistin (100 μM) was studied on the in vitro culture of rat neurons [34]. It was observed that the amyloid-beta peptide instigated the damage of mitochondrial membrane potential, nuclear DNA damage, high expression of *Bcl-2* associated X protein (BAX), p53, Caspases and lower expression of B-cell lymphoma-2 (Bcl-2) [35]. The 100 µM dose of genistin was found to be efficient against the deleterious effects of AD and restores the neuronal activity by following the caspase signaling pathways [36]. It is also reported to lower calcium overload and reverse the low fluidity of the neuronal membranes in the AD model. Free radicles were also reduced with the use of genistin and thus it is suggested for the targeting of anti-ROS (reactive oxygen species) pathway and protect the cells from death. Astrocytes are also studied for the efficiency of genistin [37]. Pretreatment of 5 μM concentration of genistin was used against astrocytes and it protects by working on interleukin 1 beta (IL-1β), tumor necrosis factor-α (TNF-α), cyclooxygenase-2 (COX-2), inducible nitric oxide synthase (iNOS) cascade, and reversing the effect of NDs in the AD model [38]. In another study, 50 μM concentration of genistin was effective and initiate IL-1β /TLR4/NF-κB/ IκB-α cascade against neuroinflammation induced by Aβ in the secondary culture of C6 [39]. It can downregulate TNF-α, IL-1β, TLR4, and NF-κB and promote the upregulation of IκB-α. Other phytochemicals are also having miraculous properties against many NDs specifically AD [40]. Genistein protects Aβ-induced neurotoxicity by inducing the PKC signaling pathway, which further regulates the activities of α- and β-secretase and thereby inhibits the formation and toxicity of Aβ [41]. Genistein also protected the hippocampal neurons against injury by calcium/calmodulin dependent protein kinase IV protein levels in AD model [42]. *Ginkgo biloba* is also considered as a neuroprotective agent. Ginkgolide A inhibits Aβ-induced neuronal degradation via JNK phosphorylation and NMDA/AMPA-induced depolarization, thus suppress the activity of plaque [43]. EGb 761 increases the neurogenesis in the adult hippocampus region and CREB phosphorylation in the transgenic mouse model of AD [44]. In one study, *G. biloba* demonstrated protection against a high dose of Bisphenol A (BPA) and played a role in the improvement of cognitive deficits [45]. A flavonoid, luteolin, would be discussed against the AD model as a possible therapeutic agent. In an in vivo study (rat model), it protects against the cognitive dysfunction which was induced by chronic cerebral hypoperfusion [46]. It is also found to be protective against high-fat diet-induced cognitive abnormalities. GSK 3α isoform is involved in the mechanistic signaling. The inactivation of GSK 3α isoform phosphorylates the PS1 which is the catalytic core of the γ-secretase complex causing a decrease in PS1-APP interaction and generation of Aβ. In literature, the role of luteolin against zinc-induced hyperphosphorylation of the protein τ in SH-SY5Y secondary cell culture is reported [47]. Luteolin is suggested as a promising candidate because of its antioxidative properties as well. In the AD animal model, luteolin slows down the escape latency and traveled distance parameters in the morris water maze while increasing the time spent in the target quadrant [48]. Melatonin modulates the proteins (i.e., GSTP1 and CPLX1) that are involved in depression and anxiety. The modulation improves the neuropsychiatric behaviors in the AD model [49]. Melatonin improves anxiety and depression-like behaviors and targets the proteomic changes in the triple transgenic AD mice model [50]. A multicenter, randomized, placebo-controlled clinical trial of 2 dose formulations of oral melatonin coordinated by the National Institute of Aging-funded Alzheimer’s Disease Cooperative Study, and it was found that melatonin was effective against AD [51]. Naringenin protects Aβ_25-35_-caused damage via ER and PI3K/Akt-Mediated Pathways [49,52]. Naringenin also protects AlCl_3_/D-galactose-induced neurotoxicity in the rat model of AD via inhibition of attenuation of acetylcholinesterase levels and attenuation of oxidative stress [53]. A new study suggested the therapeutic effects of piperine against oxidative insult, neuroinflammation, and neurochemical changes induced by ICV-STZ infusion in mice [54]. In the human brain, microvascular endothelial cells from fibrillar *β*-amyloid are protected by quercetin [55]. The flavonoid quercetin improves Alzheimer’s disease pathology and protects cognitive and emotional function in aged triple transgenic Alzheimer’s disease model [56]. in vitro AD model, resveratrol works on oxidative damage via activating mitophagy [57]. Resveratrol is found to be active against the harmful process that occurs in AβPP/PS1 mouse hippocampus and inhibit memory loss [58]. For the AD model, resveratrol is studied in randomized, double-blind, placebo-controlled, phase II trial. Resveratrol was found to be safe, effective, and can protect the BBB integrity with CNS response [59]. Thymoquinone inhibits the Aβ-induced neurotoxicity by blocking the mitochondrial dysfunction and oxidative stress [60]. S-allyl cysteine also targets the oxidative stress-induced cognitive neuronal impairment and provide possible rescue from the diseased [61]. In mice model treated with D-galactose, S-allyl cysteine involves in the alleviation of β-Amyloid, oxidative damage [62]. Eucalyptol ameliorates inflammation induced by Aβ_(25–35)_ in differentiated PC12 cells by following the NOS-2, COX-2, and NF-κB associated pathways [63]. Silibinin and silymarin administration could recover memory impairment and could decrease Aβ plaque in the brain of APP/PS1 mice [64]. By following the BDNF/TrkB pathway, Silibinin ameliorates anxiety/depression-like behaviors in amyloid β-treated rats [65]. From all the above discussion of the phytochemicals, it is imminent that researchers would be able to hit the specific targets for providing promising therapeutic relief against AD Figure 3. The details of several phytochemicals in AD protection are enlisted in Table 1.

### 3.2. Parkinson’s Disease and Parkinsonism

PD is the second most prevalent NDs of the elderly after AD, characterized by dopaminergic neuronal loss in substantia nigra pars compacta region of the brain, the striatum, locus ceruleous, raphe nuclei, nucleus basalis of meynert, and hippocampus. The main sign and symptoms of PD patients are resting tremors, rigidity, bradykinesia, muscular impairment, postural instability, difficulty in walking, soft speech, sleep problems, slow handwriting [127]. The pathological findings are the presence of Lewy Bodies (LBs, aggregation of α-synuclein) with DNA mutations in the brain of PD patients [128], with neuronal cytoplasmic inclusions in the cortex and brain stem [129,130]. The treatment of PD mainly rotates around the enhanced activities of dopaminergic neurons or inhibition of cholinergic effects [131]. Several mechanisms associated with PD accounts for oxidative stress, mitochondrial dysfunction, apoptotic pathway activation etc. [132,133] There is no cure for PD but drugs available in the market increases the life expectancy with relief in the symptoms [131]. Besides, the drug therapy demonstrates many side effects in patients [134,135]. Therefore, researchers have to treat PD at molecular and cellular levels, and use such treatments that have a multifactorial approach. Several scientists have reported plant compounds to be effective in the treatment of PD. After dissecting the oxidative stress role in the pathophysiology of PD, the phytochemicals having antioxidative properties are drawing the attention of researchers [136]. This section deals with the phytochemicals which are generally considered as antioxidative agents. In many fruits and vegetables (apples, broccoli, and onions), a polyphenolic compound is detected named quercetin. It restricts the hippocampal neuronal damage resulting in improved memory and learning (by maze test) in the rat model [137]. In one study, when quercetin was administrated to the PD model, it reversed the neuronal impairment via antioxidative pathways [138]. In PD MitoPark transgenic mouse model, quercetin protects against mitochondrial dysfunction and progressive dopaminergic neurodegeneration [66]. Quercetin also plays a specific role in the upregulation of cognitive effects in the 6-hydroxydopamine (6-OHDA) rat PD model [67]. An isoflavone, genistin is also having several health benefits including nourishment of neurons [139]. It is known as antioxidative, anti-inflammatory, and neuroprotective agent. Genistin (oral dose 10, 50, or 100 mg/kg for one week) acts as a therapeutic agent against LPS-induced cognitive impairment. These dose-dependent treatments of genistin determines to lessen memory deficits, decrease the malondialdehyde levels in the hippocampus region, increases the level of superoxide dismutase, catalase, and glutathione [140]. Moreover, genistin reformed the level of AChE activity in the hippocampus against LPS insult in in vivo model. It targets the modulation of Nrf2/NF-κB/IL-6/TNFα/COX2/iNOS/TLR4/GFAP and thus promote neuronal protection via inhibiting the neuronal inflammation and improve cognitive deficits [140]. Genistein shows the neuroprotection in the A53T mutant α-synuclein overexpressed SH-SY5Y cells [68]. Genistin protects the Transgenic *Drosophila* Model of Parkinson’s Disease against oxidative stress [69].

**Figure 4:**
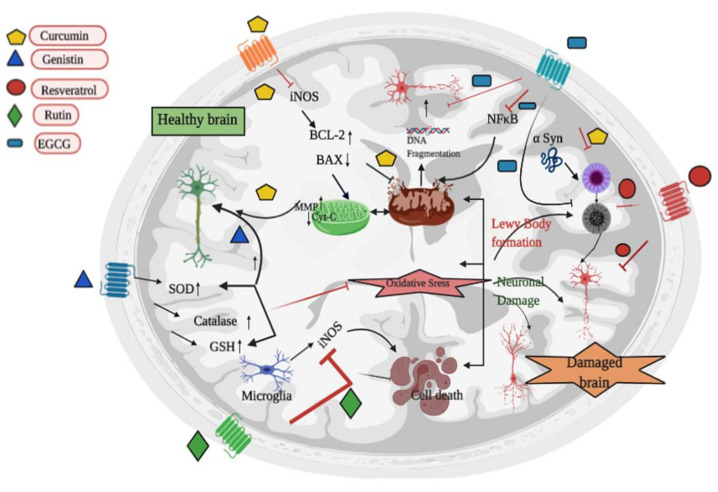
Diagrammatic presentation of Parkinson’s disease, cause, and role of phytochemicals to reverse the sign of PD. Mitochondrial dysfunction, oxidative stress, and uncontrolled cell death leads to the accumulation of Lewy bodies and resultant neuronal damage. Phytochemicals function with multiple targets. Curcumin targets the inducible nitric oxide synthase (iNOS) and regulates the ratio of BCL-2/BAX which leads to the restoration of mitochondrial function. Genistin increases the activity of SOD, Catalase, and GSH and protect the neurons from damage ignited by oxidative stress. Resveratrol inhibits the formation of Lewy body (aggregation of α-syn protein-a major cause of PD). Rutin protects the neuron by leading the anti-inflammatory pathway and EGCG inhibits the transcriptional factor NFκB and restores the normal function of mitochondria. EGCG also plays a role to inhibit DNA fragmentation. All over the activities of phytochemicals escape neurons from damage.

Other phytochemical Naringin was studied and was found to be involved in the neuroprotection by triggering the Nrf2/ARE signaling against 6-OHDA-induced neurotoxicity [141]. In in vitro study (neuroblastoma SH-SY5Y cells), it is reported to show neuronal protection by blocking the c-Jun N-terminal kinase (JNK) phosphorylation and regulation of BAX in the rotenone-induced cell line [142]. Additionally, it also inhibits the caspase-3 and PARP cleavage [143]. Naringin can lead to the anti-inflammatory pathway and provide neuroprotection in PD [70]. In in vitro PD model of SH-SY5Y cells, curcumin imparts a role in neuroprotection against toxic insult via controlling the HSP-90 [71]. Curcumin was studied in Pheochromocytoma Cell Line 12 (PC12) cells against 1-methyl-4-phenylpyridinium (MPP^+^) toxicity and fixation of mitochondrial membrane potential was found against enhancement of intracellular ROS which leads to the instigation of iNOS, and over-expression of Bcl-2 [144]. Curcumin leads to the restoration of mitochondrial membrane potential (MMP) which causes the up-gradation in Cu/Zn SOD and modulates NF-κB nuclear translocation by inhibition of interleukin-6 (IL-6) and TNF-α [145]. It was further reported that it lowers the iNOS mRNA levels and inhibits the induction of NF-κB. It is found to be effective against oxidative stress via following cytochrome c release or caspase-3 activation. In in vitro study, it is reported to act on the aggregation of synuclein inhibition and aggregation of the PD associated, A53T mutant-synuclein in SH-SY5Y cells in a dose-dependent manner [146]. By inhibiting the α-synuclein aggregation in lipopolysaccharide-induced PD model curcumin plays a role as a promising therapeutic agent [72]. Epigallocatechin-3-gallate (EGCG) is also an important bioactive component with neuroprotective potential. The mode of action of EGCG is on neuroinflammation, oxidative stress, and autophagy. Likewise curcumin, EGCG also ameliorates the mitochondrial restoration by antioxidative properties and associated signaling pathways i.e., via blocking the expression of COX-2, iNOS, and all other enzymes which causes the generation of pro-inflammatory mediators [147]. EGCG protects by antioxidative activity against 6-OHDA administration in SH-SY5Y cells (in vitro dopaminergic model) [148]. EGCG also functions as antagonistic against the NF-κB transcription factor and participates to remove the poisonous accumulation of amyloidogenic α-synuclein in many in vitro models. 1-methyl-4-phenyl-1,2,3,6-tetrahydropyridine (MPTP) treated mice also show improvement when EGCG is administrated orally [149]. EGCG also inhibits the oxidative stress in rotenone-induced in vitro model of SH-SY5Y cells [73]. In the MPTP-induced mouse model of PD EGCG helps in the modulation of peripheral immunity [74]. A natural polyphenolic compound, resveratrol, is involved in the mitochondrial biogenesis by initiating the antioxidative cascade mechanism. It also protects neurotoxic chemicals i.e., 6-OHDA and MPTP in in vitro models [150]. This natural compound shows the promising cure against cell toxicity induced by dopamine (DA) and reverse the neuronal impairment by following the cAMP/PKA pathway and thus inhibit the α-synuclein aggregation [151]. Further study also points out the SIRT1/AMPK/PGC1-α axis as a key neuroprotective pathway and provides a rationale for exploring the therapeutic potential of resveratrol in delaying PD progression [152]. By leading the CXCR4 signaling pathway, resveratrol helps in the restoration of the 6-OHDA-induced damage in the PC 12 cells [75]. Resveratrol attenuates the PD like condition via inhibition of programmed cell death through inducing MALAT1/miR-129/SNCA signaling pathway [76]. A total of 119 participants were randomized to placebo or pure synthetic resveratrol 500 mg orally once daily with a dose-escalation by 500-mg increments every 13 weeks until a final dose of 1000 mg twice daily was reached for the final 13 weeks. Visits occurred at screening, baseline, and every 6 weeks with a total resveratrol exposure of 52 weeks. Compliance with resveratrol and with placebo was confirmed by mass spectrometry of blood samples. Resveratrol may have many other molecular effects, including anti-inflammatory, antioxidant, and anti-Aβ aggregation. 480 PD patients, randomized, double-blind three dosage groups of green tea polyphenol and one placebo control group. Delay in motor function progression, increased cognition, mood, and quality of life was reported [153]. 20 double-blind, crossover, randomized, placebo-controlled phase 1 study BIA 6-512 (trans-resveratrol) 25 mg dose, 50 mg dose, 100 mg dose To study BIA 6-512 effect on levodopa pharmacokinetics when administered with levodopa/benserazide [154]. Rutin, another phytochemical act as an anti-inflammatory agent for neuroprotection which is associated with the involvement of microglia response, cytokines level, and iNOS activation [155]. Rutin facilitates protection in 6-OHDA-induced neurotoxicity in rat pheochromocytoma (PC-12) cells [77]. Rutin also targets the oxidative stress and protects dopaminergic neurons in the animal model of PD [78]. Hesperidin can be a possible promising guide for the designing of inhibitors of mutant SOD1 for therapeutic purposes. Antioxidative and anti-inflammatory properties make hesperidin reliable herbal compound against NDs. ERK/Nrf2 are key signaling molecules for following the neuroprotective pathway [156]. Neurotoxicity induced by 6-OHDA can be suppressed by the use of hesperidin in the mice model of PD [79]. Piperine treatment attenuated rotenone-induced motor deficits, and rescued the loss of dopaminergic neurons in the substantia nigra [157]. Piperine stimulates the anti-apoptotic and anti-inflammatory pathway and shows protection in 6-OHDA-induced PD rat model [80]. PD is known as the second rank on the list of NDs with poor availability of effective treatments still, the studied phytochemicals have given hope for promising and long-lasting therapy. Figure 4. The briefing of phytochemicals and their therapeutic effects on PD are listed in Table 1.

### 3.3. Amyotrophic Lateral Sclerosis

ALS also called motor neuron disease (MND) or Lou Gehrig’s disease is another NDs that is heterogeneous and both upper and lower motor neurons are degenerated causing motor symptoms. The initial symptoms vary between individuals, some exhibiting spinal-onset disease with muscle weakness of limbs, and others present with bulbar-onset disease in which they have dysarthria and dysphagia [158]. The preponderance of patients face death within 3–5 years after the first sign of disease due to respiratory failure [159]. The cause of this diseases is unknown but few cases are linked to familial history with gene mutations as a major contributor [160] and associated mechanisms of oxidative stress [161], calcium toxicity [162], inflammation [163], chronic viral infections [164], and excitotoxicity [165]. Other symptoms are spasticity, sialorrhoea, pain, muscular cramps, liver problems, deep venous thrombosis, mood alterations, respiratory insufficiency, fever, vomiting, fatigue, and nausea. In the case of ALS, the ability of the brain to convert the toxic radicals into non-toxic substances gets compromised because of mutations in *SOD1* [166]. Although, this kind of mutation does not lead to the progression of the disease but the mutation makes the *SOD1* more prone to the misfolding which causes the accumulation of protein and thus involves the reduction in the activity of motor neurons. The treatment of ALS is often symptomatic with Riluzole (Rilutek) and Edaravone (Radicava) as the only drugs approved by the FDA. These drugs can only increase the life expectancy by three to six months. Much of the efforts of physicians and other health workers is to make the quality of life (QOL) better for ALS patients. Several studies on natural plants and their active compounds are in progress to combat the dreadful symptoms of this disease. The molecular mechanisms and potential therapeutic role of the plant compounds have to be studied and implemented.

Few phytochemicals and their possible known mechanism of actions will be discussed in this section. Ginseng root shows the protective effect in *SOD-1* (G93A) transgenic mice [81]. An isoflavone, genistin can easily cross the blood–brain barrier (BBB) and attributes its role by anti-inflammatory activity via extracellular-signal-regulated kinase (ERK) and microtubule-associated protein kinase (MAPK) signaling in the microglia which leads to the neuronal inflammation. The neuroprotective role of genistin was detected in the SOD1-G93A mice [82]. Ginseng roots inhibit the onset and increase the survival of affected cells delays the onset and prolongs the survival of affected cells [167]. The most promising phytochemical, curcumin has a protective role in ALS too. It acts for mitochondrial biogenesis leading the AMP/PGC-1α and/or Nfr2 pathway [83]. The clinical studies show the effectiveness of curcumin for slowing down the progress of the disease. Curcumin helps in the improvement of aerobic metabolism and oxidative stress. The antioxidative mechanism is responsible for leading the protective effect against ALS [83]. A studied phytochemical named withaferin A shows an anti-inflammatory effect by working as antagonistic of NF-κB and is found to be effective for the improved motor activity in transgenic mice model [84]. ALS mouse model generated by the point mutation in SOD1 was studied and it was observed that by the withaferin treatment there was a late progression of the disease and more survival time in comparison to the non-treated ALS mice. The root extract of *Withania* was also tested against mutated transgenic mice (TDP-43A315T mice) and manifested with removal of abruptly cytoplasmic motor neurons, reduction in inflammatory markers, and improvement in cognitive performance [168]. It is reported that ALS is following numerous pathways therefore, combined therapy could be more efficient for targeting more than one signaling pathway within a single attempt. EGCG has a power of antioxidation, hampered activation of caspase-3, NF-κB and thus reduces microglial activation resulting in decreased neuroinflammation and increased neuronal survival [169]. EGCG stimulates the iNOS/NF-κB/ Caspase-3 and shows the increased number of neuronal cells, lower microglial activation in transgenic mice model [85]. One more phytochemical named celastrol also reveals the anti-inflammatory, antioxidative property. Celastrol exhibits an antioxidative effect in SOD1G93ANSC34 cells by leading MEK/ERK and PI3K/Akt signaling pathways [86] Celastrol protects the H_2_O_2_-induced oxidative stress model by activating the antioxidative pathway in ALS model [86]. The bran oil treatment helps in the significant enhancement of antioxidative enzymes and suppress the effect of ROS and malondialdehyde (MDA) in rotenone-induced ALS model [170].

ALS study was also conducted in bone marrow-derived mesenchymal stem cells (MSCs) by activating the SIRT1/AMPK pathway and it is noted a significant decrease in the ALSMSCs compared to normal healthy control originated BM-MSCs by using resveratrol. Neuro-progenitor markers were increased in resveratrol treated ALS-MSCs [87]. All these phytochemicals along with the ones enumerated in the table could be promising therapeutic agents which can be the best alternative of commercial drugs with negligible side effects Table 1.

### 3.4. Huntington’s Disease

HD is an annihilative, inherited, and familial illness, with progressive loss of brain and muscle functions. This is a single-gene disorder in which there is programmed degeneration of neurons within different regions of the brain. In wild type gene, there are 6-35 CAG repeats in exon 1 of the *Huntingtin (Htt)* gene on chromosome number 4 whereas, in the affected persons, this repeat increases to more than 36 [171]. As a result of this, there is an accumulation of Huntingtin protein in the neuron causing its death. Almost 95% of GABAergic medium spiny neurons (MSNs) projecting to substantia nigra and globus pallidus are lost causing atrophy of cortex, thalamus, and hypothalamic nuclei. The symptoms include concentration problems, short-term memory, tumbling, lack of focus, clumsiness, depression weight loss, feeding problems, difficulty in speech, uncontrolled face movements, itching etc. [172]. There is a lot of research going on in the field of HD prevention and treatment. Many experimental models have been experimentally validated to test the efficacy of different drugs. Hitherto, there is no cure for the disease, drugs available only give symptomatic relief. There have always been natural compounds linked to various diseases and a lot of them have shown promising effects in preclinical studies.

One of the major factors for the progression of HD is the abrupt function of mitochondria [173]. The activity of complex II of the respiratory chain gets reduced in the affected brain region. Protopanaxtriol limits the overproduction of free radicals and attenuates the expression of heat shock protein 70 (Hsp-70), along with the restoration of SOD activity inhibiting the formation of free radicals. Protopanaxtriol enhances the Nrf2 entry to the nucleus and increases the expression of Heme oxygenase-1 (HO-1) and NAD(P)H dehydrogenase [quinone]1(NQO1) [88]. Melatonin also has been shown to reduce neural damage in PD, AD as well as in ischemia-reperfusion injury against d-aminolevulinic acid and a variety of other neural toxins. In the case of mitochondrial injury, induced by 3-nitropropionic acid there was a restoration by the treatment with melatonin by leading the antioxidative pathway [88,174]. Melatonin also rescues the HD animal model from the oxidative stress induced by 3-nitropropionic acid [89]. Resveratrol attenuates the expression of ATG4 and permits the lipidation of LC3 and promotes the mortification of the polyQ-Htt accumulation and saves the neuronal cells against dopamine-induced toxicity [90]. EGCG inhibits the accumulation of misfolded proteins and oligomerization of mutant Htt exon 1 *protein* in vitro, which indicates the early interference of EGCG in the misfolded protein accumulation [175]. In another study, EGCG reduced the cytotoxicity and polyQ-mediated htt protein aggregation in a yeast model of HD [176]. EGCG attenuates the adverse effect of misfolded huntingtin protein in early-stage in HD model [91].

**Figure 5:**
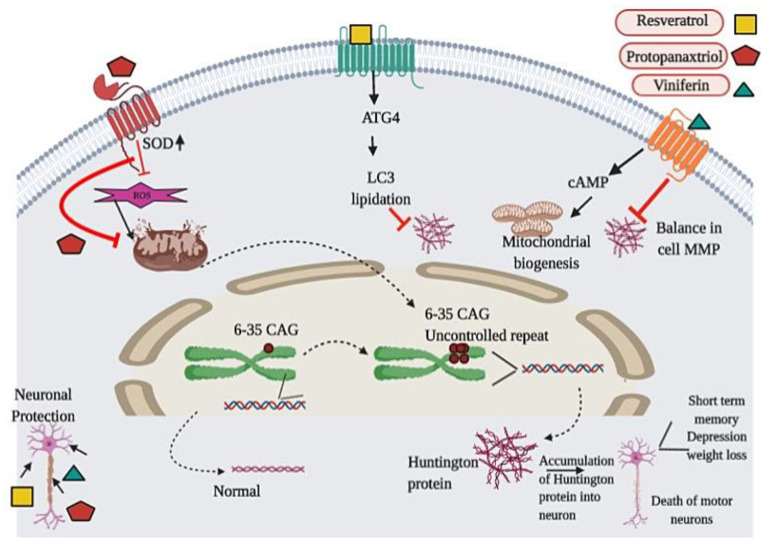
Diagrammatic presentation of Huntington Disease progression and protection of neurons with phytochemicals: 6-35 CAG repeats lead to the formation of Huntingtin protein which consequently accumulates in the neurons and damage motor neurons. In the early stage, damaged motor neurons result in short term memory while on a later stage, this condition changes into depression, weight loss, and many more. The studied phytochemicals generally work against free radicals and restrict the accumulation of Huntingtin protein in the cells. This inhibition further leads to neuronal survival. Resveratrol works on LC3 lipidation while protopanaxtriol and viniferin leads to the antioxidative pathway and restore the function of mitochondria to protect the neurons and stop further accumulation of Huntingtin protein in neurons.

One more phytochemical, trans-ε-Viniferin (viniferin), was also found to be effective against mutant *Htt*-induced depletion of SIRT3 and thus save the cells from a mutant protein [177]. Viniferin also plays a crucial role in reducing the level of free radicals and thus participate in the protection of MMP in the cells having mutant *Htt*. The presence of mutant *Htt* indicates the lower deacetylase activity of SIRT3 which leads to decreased cellular NAD levels with the biogenesis of mitochondrial cells [178]. Viniferin enhances the mitochondrial biogenesis by activation of AMP-activated kinase [92]. It was reported that systemic 3-NP administration significantly enhances the level of lipid peroxidation (LPO), nitrite and lactate dehydrogenase (LDH), downregulate the antioxidant enzyme (superoxide dismutase and catalase) levels, and inhibit the synthesis of ATP by blocking the activity of mitochondrial complex in the different regions (striatum and cortex) of the brain [179]. Figure 5. The root extract of *Withania somnifera* is used against 3-NP-induced gait abnormalities, mitochondrial dysregulation, and oxidative stress, in striatum and cortex of the rat brain, in vivo model [93]. The detailed functions of phytochemicals are well explained in Table 1.

### 3.5. Vascular Cognitive Impairment

Vascular cognitive impairment is distinguished by a specific cognitive profile involving preserved memory with impairments in attentional and executive functioning [180]. Vascular dementia should be broadened to recognize the important part cerebrovascular disease plays in several cognitive disorders, including the hereditary vascular dementia, multi-infarct dementia, post-stroke dementia, subcortical ischemic vascular disease and dementia, mild cognitive impairment, and degenerative dementias (including Alzheimer’s disease, frontotemporal dementia, and dementia with Lewy bodies). Alzheimer’s disease (AD) and vascular dementia (VD) share key pathologies including oxidative damage, oral supplement of phytochemical medicines, which are well-known for their antioxidant properties, can be a viable therapy for both types of dementia. In this study, the therapeutic potential of the *Aster ageratoides* extract (AAE), was found to be effective in experimental rat models of AD and VD. These results provided evidence that AAE supplements can exert anti-AD and -VD efficacies and suggested that AAE might be used as an edible phytotherapeutic for the two major types of dementia [94]. Another study suggested that bilobalide (BB) protected against learning and memory impairments, neuronal apoptosis, and oxidative stress in a rat model of AD induced by Aβ_25–35_ peptide. Additionally, the inhibition of TNF-α and Aβ_1–40_ expression is also involved in the action mechanism of BB in this experimental model. The results indicated that BB has protective effects on Aβ_25–35_-induced learning and memory impairments and neuronal oxidative damage [95]. *Ginkgo biloba* extract (EGb 761) is widely used to treat cerebral disorders. EGb761 increases proliferation of neural stem cells in the subventricular zone and dentate gyrus, and significantly improves learning and memory in rats with vascular dementia. Studies reported the effects on endogenous NSCs in rat models of vascular dementia and NSCs in the mouse cochlear. *G. biloba* seemed to slow down the cognitive deterioration in patients with VCI, but the effect was shown in only one of the four neuropsychological tests administered [96]. In a randomized, double-blind, placebo-controlled clinical trial *G. biloba* standardized extract showed effectiveness against vascular cognitive impairment [97]. Huperzine A is a new type of *Lycopodium* alkaloid monomer isolated from *Huperzia serrata*. It inhibits acetylcholinesterase activity and increases levels of acetylcholine in the brain thereby improving cognitive function in patients with dementia [98]. A total of 92 participants were included in the two studies, with 46 in the Hup A group and 46 in the control group. The number of patients in the individual studies ranged from 14 to 78, and the duration of trial ranged from 12 to 24 weeks [99].

Stroke is itself not a neurodegenerative disease but can lead to a secondary neurodegeneration. It has been known to be a major cause of VCI. It is the third major cause of death in brain disorders worldwide [181]. There is a sudden cessation of transient or permanent reduction of the blood flow to a particular area in the brain leading to deficiency of oxygen, glucose, and energy. The main region affected in the case of Ischemia is cornu ammonis 1 (CA1) of the hippocampus [182]. There is the death of pyramidal neurons of the CA1 region, delayed to 4 to 5 days of ischemia, hence the name is “delayed neuronal death” (DND). It is followed by a series of pathological processes working together including excitotoxicity, oxidative stress, apoptosis, inflammation, gliosis of astrocytes, and microglia in the CA1 region after ischemia/reperfusion. There is a huge burden of the depletion of the endogenous antioxidants and loss of mitochondrial function engendering neuronal death via intrinsic programmed cell death causing behavioral and histological alterations [183]. All this is followed by the breakdown of BBB causing edema. Edema activates the secretion of proinflammatory cytokines like TNF-α, IL-1β, and IL-6 by activated immune cells [184]. The oxidative stress pathway along with the inflammatory and apoptotic pathway causes further loss of neurons and brain tissues expanding the infarct area [184]. The treatment of this dreaded disease may lie in the science of phytochemicals. Many studies showing prevention against stroke have been extensively published, some of them are discussed below.

**Figure 6:**
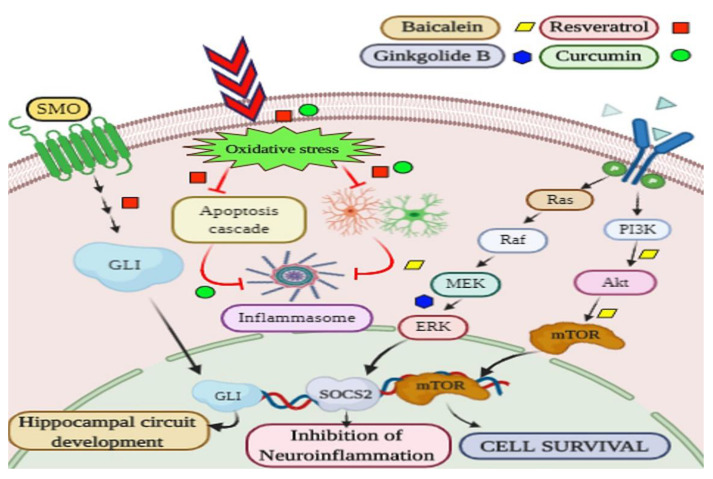
Overall pathogenesis of neuronal loss in ischemic stroke and their prevention by phytochemicals. Oxidative stress, found in the neurons of the ischemic brain is prevented by resveratrol and curcumin. These phytochemicals also prevent the inflammation and apoptosis induced by oxidative stress. Baicalein prevents the formation of inflammasome reducing inflammation and edema. Resveratrol activates SMO/GLI pathway leading to the development of hippocampal neuronal circuit. It also activates the PI3K/Akt/mTOR pathway leading to cell survival. Another phytochemical ginkgolide B acts on MEK/ERK/SOCS2 pathway causing inhibition of inflammation in neurons.

Resveratrol has been used in one of the studies where it is reported that pretreatment of resveratrol improved the neuronal functions by lowering the chances of ischemic injury in in vivo model. Additionally, it also inhibits axonal deterioration and promotes neuronal growth and synaptic formation. In in vitro study, it is found to be effective against oxygen-glucose deprivation/reoxygenation (OGD/R) injury and promoted neural stem cell (NSC) proliferation [100]. SIRT 1 is involved in the Shh signaling arbitrated the effects of resveratrol on the OGD/R injury neuronal model [100]. Its administration leads to neuronal survival, reticent the apoptosis, and synaptic formations with the upregulation of SIRT1. In rat stroke model, resveratrol slows down the ischemic injury and upgraded the neuronal functions by Sonic Hedgehog Signalling [101]. Ginkgolide B stimulated the proliferation and differentiation of neural stem cells in the in vivo models of NSCs in the rat brain following cerebral ischemia, thereby increasing the proportion of neurons to improve neurological outcomes [102]. Ginkgolide B is a potential neuro-protectant and in a study, the cellular and molecular mechanisms were dissected out by following the BDNF, EGF, and Suppressor of cytokine signaling 2 (SOCS2) [185]. Another phytochemical, curcumin, extensively studied shows beneficial effect in case of stroke too. The role of inflammation is the key factor in raising the problem of the ischemic stroke. Curcumin has shown the neuroprotective effect in the in vivo model of brain ischemia by showing anti-inflammatory properties [186]. It has also shown protection against ischemic injury in N2a cells and mouse brain with stroke [103]. Curcumin plays a significant role in neuronal survival and proliferation in the model of global brain ischemia [104]. EGCG, another phytochemical, also can remove the free radicals by increasing the antioxidative enzyme level and also downregulating the MMP-9. It inhibits the neuronal damage in transient focal cerebral ischemia instigated by middle cerebral artery occlusion (MCAO) in mice. It follows the mTOR-AMPK pathway in stressed ER and impaired the oxidative stress responses and enhance autophagy-dependent survival. It also promotes cell multiplication, differentiation, neovascularization, tube formation, and cell homing by associating the vascular endothelial growth factor (VEGF), regulation of BAX, Bcl-2, LC3B, caspase-3, mTOR and Beclin-1 [187]. It has also protected HBMVECs from Ischemia/Reperfusion injury by ameliorating apoptosis and autophagy and promoting neurovascularization [105]. EGCG has a protective effect on rat brain injury induced by MCAO, possibly by modulating the PI3K/Akt/eNOS signaling pathway [106]. Baicalein also has shown a promising effect in the subacute phase of cerebral Ischemia/reperfusion (I/R) injury in a rat model of ischemia-induced by occlusion of the middle cerebral artery (MCA). Furthermore, it is showed that baicalein inhibits the expression of NF-κB by reducing the IκBα phosphorylation and nuclear translocation of NF-κB/p65, this cascade of signaling molecules is associated with the down-regulation of the pro-inflammatory factors IL-6, IL-18, and TNF-α [107]. Additionally, baicalein inhibits the phosphorylation of ERK, p38, and JNK. These key molecules were involved with the modulation of microglia/macrophage M1/M2 polarization. Studies show the antioxidative activity by decreasing the expression of caspase-3 along with increasing the Bcl-2/Bax ratio, thus protecting the neurons. Baicalein follows the PI3K/Akt/mTOR signaling pathway via decreasing the LC3-II/ LC3-I ratio [188]. Figure 6. *Ligusticum chuanxiong* is used as a therapy for cerebrovascular and neuronal diseases. It works against OGD-reoxy-induced injury and can induce protective HSP-70 expression via the activation of the MAPK pathway [108]. Sinomenine, a natural phytochemical shows the neuroprotection by behaving as an anti-inflammatory and anti-apoptotic agent in a mouse model of middle cerebral artery occlusion (MCAO) and astrocytes/microglia treated with oxygen-glucose deprivation (OGD). It was observed that OGD downregulates the p-AMPK and at the same time activates the inflammasome NLRP3 in mixed glial culture (in vitro model) [109,189]. Honokiol, the main biphenyl neolignan, also shows the antioxidative effects, anti-inflammatory, anti-apoptotic activity. It helps to reduce the cerebral infarction [190]. Honokiol attenuates the inflammatory reaction during cerebral ischemia-reperfusion via inhibiting the by NF-κB activation and cytokine production of glial cells [110]. Zingerone targets the oxidative stress and provide protections in focal transient ischemic rats model [111]. Zingerone (4- (4-hydroxy-3-methoxyphenyl)-2-butanone) is an innocuous, easily available, and economically cost-effective compound with promising therapeutic effects. Having anti-inflammatory, antispasmodic, antioxidative properties, zingerone protects against oxidative stress and damage. It leads to the antioxidative pathway for protection, thus help against ischemic insult [111]. Perillyl alcohol ameliorates the condition of ischemia-reperfusion injury via inhibition of the oxidative damage by following the NF-κB, COX-2, and NOS-2 associated pathway in in vivo Rat model [112]. It is also reported that piperine vanquishes the cerebral ischemia–reperfusion-induced inflammation anti-inflammatory pathway i.e., NOS-2, COX-2, and NF-κB [113]. The list of phytochemicals and the associated mechanisms are explained in Table 1.

### 3.6. Prion Disease

Prion disease is also known as transmissible spongiform encephalopathies (TSE) and is the only naturally occurring infectious protein misfolding disorder. Human prion disease is caused by the unnatural conversion of normal prion protein (PrP^c^) into an abnormal form of a protein called prions (PrP^sc^), it stands for pertinacious infectious particles [191]. PrP^c^ is a surface glycoprotein expressing in multiple tissues, however, it is highly expressed in the CNS [192]. The onset and progression of the prions disease are explained by the “protein only-hypothesis”, wherein it is understood how a protein self-replicates without a nucleic acid. It claims that PrP^c^ itself is the infectious agent [193]. During the progression of prion disease, the infectious protein PrP^sc^ aggregates and becomes resistant to proteinase digestion. This is followed by PrP^sc^-seeded conversion of PrP^c^ into PrP^sc^, therefore, resulting in the high accumulation of PrP^sc^ in an individual, which leads to neurodegeneration and the clinical manifestation of the disease [194]. This disease is classified as rare both in the progression and manifestation [195].

Several studies have been done to identify the potent agents for drug development for Prion disease. In a recent study, synthetic human prion protein (PrP 106-126) was used as an inducer to manifest prion diseases like signaling. Baicalein from *Scutellaria biacalenesis* attenuated ROS production and also inhibited ROS-induced mitochondrial dysfunction in PrP 106-126-induced-SH-SY5Y. JNK plays an important role in PrP 106-126-induced neuronal apoptosis in SK-N-SH cells, however, baicalein inhibited the JNK signaling in SK-N-SH cells [114]. Resveratrol, a flavonoid with several bioactivities is also known to prevent the PrP 106-126-induced neurotoxicity in SH-SY5Y and SK-N-SH cells by activating autophagy. Thus preventing the mitochondrial dysfunction, apoptosis, and neuronal cell death [115]. Autophagy is a cellular mechanism opted by several phytochemicals to protect against PrP-106-126-induced neurotoxicity, hinokitiol is a monoterpene that activates autophagy and it inhibits apoptosis through stabilization of hypoxia-inducible factor (HIF)-1α, which is important for the regulation of oxygen homeostasis [116]. Rutin has been found to prevent the PrP 106-126-induced neurotoxicity in mouse hippocampal cell line (HT22). Their results showed that rutin was able to increase the expression levels of neurotrophic factors like BDNF, glial cell-derived neurotrophic factor (GDNF), and nerve growth factor (NGF). They also observed the inhibition in apoptosis through suppression of FASL, FAS, caspase 8, and by inhibiting the activity of caspase 3 [117]. Table 1 enlist various neuroprotectant phytochemicals used in the case of Prion disease.

### 3.7. Frontotemporal Dementia (FTD)

FTD is a term that encompasses a broad group of heterogeneous progressive neurological syndromes. These are non-Alzheimer dementias characterized by the atrophy in the frontal and temporal lobe [196]. As frontal and temporal lobes are highly affected in this disease, the functions controlled by these areas are the outcome of FTD such as behavior and language. Some people with FTD have drastic behavioral and personality changes that make them socially inappropriate and impulsive whereas some people lose the ability to use language [197]. Some other common symptoms include lack of inhibition, apathy and judgment, repetitive compulsive behavior, decline in personal hygiene, eating the inedible objects, and loss of sensitivity towards another person’s feelings [198]. FTD can be categorized into three common variant syndromes; behavioral variant FTD (bvFTD), non-fluent/agrammatic variant primary progressive aphasia (nfvPPA), semantic variant PPA (svPPA) [199]. A subtype of FTD is Pick’s disease (PiD) which is a neurodegenerative condition that results in irreversible dementia where an excessive amount of tau inclusions known as Pick bodies is a key pathological feature [200]. Pick bodies are round argyrophilic neuronal inclusions. PiD causes cortical atrophy affecting frontal and temporal poles including limbic systems, neocortex, and dentate granular cells of the hippocampus. Such neuronal degenerations result in behavioral changes/alterations and personality disorders [201]. The early signs for PiD are apragmatism and mutism. Some studies refer to PiD as Niemann-Pick disease (NPD) based on the name of the scientist who reported the first case [200].

Not many phytochemicals have been explored for their effect on FTD. Nicotine, a plant alkaloid has been found to have a correctional effect on the behavior of *Grn*^-/-^ (progranulin gene) mice. Haploinsufficiency of progranulin levels is the result of any mutation in the Grn gene, which is one of the cases of FTD [118]. Mice with insufficient amounts of progranulin secrete inflammatory cytokines at elevated levels and develop microgliosis [202,203]. This study reported that daily intervention of nicotine reversed the sociability and improved the behavioral change in the *Grn*^-/-^ mice [202]. A traditional Chinese herbal medicine known as Yi-Gan San also known as Yokukansan in Japan containing a mixture of seven dried herbs *Atractylodes lancea, Cnidium officinale, Uncaria rhynchophylla, Angelica acutiloba, Poria cocos, Bupleurum falcatum*, and *Glycyrrhiza uralensis* in a ratio of 4:3:3:3:4:2:1.5 [204] was found to alleviate behavioral changes in patients with FTD [205]. Extracts of *Piper nigrum* (fruit) and *Curcuma longa* (rhizome) also inhibited the AChE significantly which is considered one of the key targets for therapeutic strategies against dementia [119]. The nanoformulation of curcumin can be a promising agent against Niemann-Pick disease type C astrocytes [120]. Curcumin has been reported to have several medicinal properties, wherein it was observed that nanoformulations of curcumin were able to increase cytosolic calcium ion levels and cell viability in astrocytes [120]. Another compound δ-tocopherol was found to decreased cholesterol levels and also lipid accumulation, it also reduced lysosomal enlargement. It was also reported to increase intracellular Ca^2+^ levels [121]. A study by Nekohashi et al., reported that quercetin and luteolin significantly decreased the cholesterol levels as cholesterol [122] as cholesterol is one of the key characteristics of PiD. The details of phytochemicals are given in Table 1.

### 3.8. Spinocerebellar Ataxia (SCA)

SCA is a term used to refer to hereditary (autosomal dominant) ataxias, these are neurodegenerative conditions affecting the cerebellum (movement controlling) part of the brain and the spinal cord [206]. It comprises a large group of neurodegeneration characterized by cerebellar ataxia with oculomotor dysfunction, dysarthria, also affecting the brain stem. The resulting ataxia is an outcome of atrophy in the cerebellum [207]. All types of SCA are associated with the loss of coordination in eye and hand movements including speech. SCA usually has an adult-onset and the progression is gradual, therefore worsening with time [208]. To date, 44 types of SCA have been identified and classified as SCA1 through SCA44. Many SCAs are an outcome of CAG nucleotide repeat expansion encoding polyglutamine, resulting in the involvement of toxic polyglutamine protein (polyQ). The most recent genetic cause of SCA44 reported in 2017 [209].

The focus on nutraceuticals against this disease is not that much, however, trehalose is a natural compound abundantly found in invertebrates, plants, and microorganisms. Studies have shown that it has a neuroprotective effect exhibited through interaction with protein folding. Analog of trehalose, melibiose has been reported the combat neurotoxicity/neurodegeneration by upregulating autophagy [123]. Melibiose significantly inhibited the aggregation of polyQ decreased ROS levels. It also displayed potential in decreasing SCA17TBP/Q_79_ and significant inhibition to Purkinje cell aggregation in SCA17 transgenic mice [124] Table 1.

### 3.9. Spinal Muscular Atrophy (SMA)

SMA is a genetic disorder characterized by the atrophy in skeletal muscle. The onset of SMA takes place due to the loss of motor neurons that regulate muscle movement. It results in weakness and makes the movement or any physical activity challenges, this condition worsens with time [210]. This disease is caused by the inadequate production of survival motor neuron (SMN) which is mainly expressed by gene SMN1. SMN2 is also responsible for the production of SMN but relatively far less. [211]. Mutations in SMN1 such as alter or loss results in observed in patients with SMA [212]. The presence of additional third or more copies of the SMN2 gene partially compensates for the damage caused due to the SMN1 gene and milder symptoms are seen in patients [213]. It mainly affects infants and restrict physical movement. SMA is categorized into five types (0-IV) based on the age of the patient and the severity of the disorder [214].

Since SMA is a genetic disorder, the plant products that can reverse or correct the damage are to be identified. *Brucea javanica* and its major compound as found to be bruceine D were found to have SMN2 splicing-correcting property. This study showed that they corrected the splice defect and also increased the activity of SMN2 significantly. *B. javanica* was able to ameliorate the muscle defects [125]. Triptolide is another phytochemical that showed a significant effect in combating SMA in SMA-like mice. The results showed that triptolide isolated from *Tripterygium wilfordii* elevated the production of SMN protein and transcription of SMN2 both in vivo and in vitro experimental models. Hsu et al. concluded that triptolide is a modulator of SMN expression. Triptolide enhances the expression of transcript and protein levels of survival motor neurons in human SMA fibroblasts and improves survival in SMA-like mice [126] Table 1.

## 4. Conclusions

This review focuses on the use of phytochemicals as a preventive approach to neurodegenerative disorders as they are cost-effective, readily applicable, and easily accessible compounds with a promise in future use. On the other hand, there have always been limitations to the clinical use of these phytochemicals. The main issue focuses on the bioavailability and digestibility of these natural compounds in the body of the patients. Blood–brain barrier is another hurdle in the use of phytochemicals for problems related to the nervous system. Moreover, it is time-consuming therapy for effectiveness against the disease.

Natural products possess variable neuroprotective properties, which can be exploited to target ND therapeutically. Phytochemicals have multi-targeted applications with numerous advantages over currently targeted drugs on the market and have limited adverse reactions. They can also help in developing food supplements and tonics from the edible plants possessing neuroprotective activity. However, given the complexities and heterogeneity of phytochemicals, a cautious approach is necessary to define herbal mixtures and compositions of different compounds for treatment purposes. Keeping this in mind, more preclinical and clinical studies are being conducted. Finally, these plants and other remaining body of studies can very well be executed in not only designing safer drugs to combat the NDs but also preventing the onset. Consequently, this review will abet the researchers for further extensive study in this field.

## Figures and Tables

**Table 1:** Role of various phytochemicals in different neurodegenerative diseases with possible mechanisms of action with special reference to in vitro, in vivo and clinical studies.

Disease	Plant	Natural Compound	In-Vitro/In-Vivo Models/Human Trial	Dose & Route	Mode of Action		References
**Alzheimer’s Disease**	*Curcuma longa*	Curcumin	Acrolein exposed HT22 murine hippocampal neuronal cells	5 μg/mL/30 min	↓ AD-like pathologies, MDA level, ↑ levels of GSH, SOD, ↓ metalloprotease, APP, β-secretase, ↑BDNF/TrkB signaling		[27]
			p25 Transgenic Mouse model	4 g/kg for 12 weeks; po	↓ tau, amyloid accumulation, ↑ proinflammatory cytokines		[29]
			Pilot study with curcumin and ginkgo on 34 possible or probable AD patients	1 g/day, 4 g/day 120 mg/day ginkgo leaf extract for 6 months	No significant difference in Mini-Mental State Examination scores or plasma A*β*40 levels between 0 and 6 months. No side effect of curcumin		[30]
		Longvida	40 subjects: AD, MCI, healthy	20 g/day for 7 days	Retinal imaging of Aβ plagues differentiated between AD and non-AD subjects		[31]
	Many plants	Apigenin	Copper induced SHSY5Y cells	10.0 mM for 24 h	↑antioxidation, mitochondrion protection, ↑MAPK signaling		[32]
			APP/PS1 double transgenic mouse model	40 mg/kg for 5 day/week	↑behavior, ↓Aβ burden, ↑ERK/CREB/BDNF pathway, ↓oxidative stress		[33]
	*Glycine max*	Genistein	Rat hippocampal neuronal cells	0.4 μg/mL	↑ α-secretase, ↓ β-secretase, ↑PKC signaling pathway		[41]
			Bilateral hippocampal Aβ_25–35_ injected rat Model	90 mg/kg for 42 days	↓ p-tau, CALM, CAMKK1, p-CAMK4 protein, escape latency		[42]
	*Ginkgo biloba*	Ginkgolides A or B	Mouse primary cortical neurons	100 or 300 µM/1.5 h	↓ AMPA- and NMDA-induced depolarization, ↓JNK phosphorylation		[43]
			TgAPP/PS1 mice	100 mg/kg for 1 month	↑ cell proliferation in the hippocampus, ↓ Aβ oligomers and ↑ pCREB levels		[44]
	Many plants	Luteolin	ICV-STZ rat model	10 and 20 mg/kg	↑ escape latency, ↑ thickness of CA1 pyramidal layer		[48]
	Many plants	Melatonin	murine N2a neuroblastoma cells	10 µM	↑ cell viability, ↓ cell blebbing and retraction, ↓ LPO, intracellular Ca^+2^		[49]
			3xTg-AD transgenic mice	10 mg/kg/day in drinking water for 1 month	↑ anxiety and depression like behaviors, ↑ GST and complexin-1		[50]
			157 AD patients	10 mg melatonin	↑ nocturnal total sleep time, ↓ wake after sleep		[51]
	*Citrus junos*	Naringenin	Aβ_25-35_ treated PC-12 cells	0.4 µM for 2 h	↑ cell viability, ↑ ER-mediated PI3K/Akt signaling pathway, ↓ caspase-3		[52]
			AlCl_3_+D-gal rat model	50 mg/kg for 2 weeks po	↑ behavioral parameters, ↑ antioxidant enzymes, ↓ LPO, AChE, ↑ ACh levels, 5-HT levels and DA levels, ↓ DOPAC levels		[53]
	*Piper nigrum*	Piperine	ICV-STZ C57BL/6 mice	10 mg/kg/22 days	↑ behavioral parameters, ↑ antioxidant enzymes, ↓ LPO, ↑ ARG1, CD206, NE, DA, 5-HT and GABA levels and ↓ glutamate level in hippocampus, ↓ CD86 and iNOS,		[54]
	Many plants	Quercetin	fAβ 1-40 insulted hBMECs	0.3, 3 and 30 µmol/L	↑ cell viability, ↑ γ-GT and ALP, ↓oxidative stress, ↑ barrier function		[55]
			triple transgenic AD (3xTg-AD) mice	25 mg/kg every 2 days for 3 months	↑ neuronal population, ↓ β-amyloid accumulation, tau accumulation, astrogliosis and microgliosis, ↑behavior parameters		[56]
	Grapes and other plants	Resveratrol	Aβ_1-42_-treated PC12 cells	3 μM	↑ cell viability, ↑ mitophagy, ↓ apoptosis and ROS		[57]
			AβPPswe/PS1dE9mouse model	16 mg/kg/day	↑ behavior, ↓ plague pathology, ↑ p-AMPK/LKB pathway, ↓ SIRT-1, ↑ IL1β and TNF		[58]
			199 patients’ mild to moderate dementia due to AD.	500 mg once daily with 500 mg increments every 13 weeks leading to 1000 mg twice daily.	↑ CSF and plasma Aβ 40 in placebo, preserves brain barrier integrity		[59]
	*Nigella sativa*	Thymoquinone	Aβ_25-35_-treated PC12 cells	4 μM for 24 h	↑ cell viability, ↓ LDH levels, ↓ TBARS, ↑ GSH, antioxidant enzymes, ↓ ROS, AChE, NO levels, ↑ MMP, ↓ iNOS expression		[60]
	*Allium sativum*	s-allyl cysteine (SAC)	ICV-STZ mice model	30 mg/kg i.p. for 15 days	↑behavior, ↑ GSH, antioxidant enzymes, ↓ LPO, ↑ Bcl2 and ↓ p53 levels		[61]
			D-galactose (DG) mice	1 g/L in drinking water for 7 weeks	↓ Aβ1-40 and Aβ1-42, ↓ APP and BACE1 expression, ↑ PKC activity		[62]
	Many plants	Eucalyptol	Aβ_25-35_-treated PC12 cells	10 μM/24 h	↓LDH levels, ↓ TBARS, ↑ GSH, antioxidant enzymes, ↓ ROS, AChE, NO levels, ↓ cytokines, ↓ NF-κB, COX-2, iNOS, ↑cell viability		[63]
	*Silybum marianum*	silibinin	APPswe/PS1dE9 double-transgenic mice	100 mg/kg/15 days	↑ behavior in MWM, ↓ amyloid plaque burden, ↓ gut bacterial		[64]
			Aβ1-42 model of rats	25, 50 and 100 mg/kg for 10 days	↓ depression, anxiety behavior, ↓ neuronal damage, ↑ BDNF and TrkB expression, ↑ autophagy		[65]

**Parkinson’s Disease**	Many plants	Quercetin	The mouse dopaminergic MN9D cell line	10 and 30 µM quercetin for 24 h	↑ PKD1 pro-survival signaling, Akt and CREB phosphorylation, BDNF expression, mitochondrial biogenesis		[66]
			6-OHDA model of rats	100, 200, 300 mg/kg for 14 days	↑ motor and non-motor deficits, antioxidant enzyme activities, ↑ neuron density in hippocampus, ↓ AChE		[67]
	*Glycine max*	Genistein	SH-SY5Y cells overexpressing A53T mutant α-synuclein	20 μM for 24 h	↑ cell viability, ↓ MDA content, ↑ GSH, ATP and Na^+^K^+^ATPase levels, ↓ apoptosis		[68]
			Transgenic Drosophila Model expressing human α-synuclein	10, 20, 30, and 40 µM for 24 days	↑ life expectancy, ↓ loss of climbing ability, ↓ oxidative stress, ↑ dopamine content		[69]
	Grapefruit and other citrus fruits	Naringin	MPP^+^-induced Parkinson’s disease rat model	80 mg/kg for 4 days	↑ GDNF expression and mTORC1, ↓ TNF-α		[70]
	*Curcuma longa*	Curcumin	MPP+-induced SH-SY5Y cell	40 µM for 24 h	↑ cell viability, DA, Bcl-xl level, ↓ caspase 3, Bax level and HSP-90 levels		[71]
			Lipopolysaccharide-induced PD rat model	40 mg/kg i.p. for 21 days	↓ GFAP, NFκB, TNF-α, IL-1β, IL-1α, iNOS, oxidative stress, α-synuclein aggregates, apoptotic markers		[72]
	Green tea	Epigallocatechin gallate (EGCG)	Rotenone treated SH-SY5Y cells	25 or 50 μM for 24 h	↓ caspase-3 and apoptosis, ↑ cell viability, SOD		[73]
			MPTP mouse model	25 and 50 mg/kg for 20 days	↑ behavior, TH-positive neurons, ↓ TNF-α and IL-6		[74]
			480 PD patients, randomized, double blind	three dosage groups of green tea polyphenol and one placebo control group	Delay in motor function progression, ↑ cognition, mood and quality of life		ClinicalTrials.gov identifier: NCT00461942
	Grapes and other plants	Resveratrol	6-OHDA treated PC 12 cells	50 μM for 24 h	↑cell viability, MMP, ↓ apoptosis, CXCR4 protein levels		[75]
			MPTP mouse model	50 mg/kg for 3 weeks	↑ TH+ cells and miR-129, ↓ MALAT1, SNCA and apoptosis		[76]
			20 double blind, crossover, randomized, Placebo controlled phase 1 study	BIA 6-512 (trans-resveratrol) 25 mg dose, 50 mg dose, 100 mg dose	To study BIA 6-512 effect on levodopa pharmacokinetics when administered with levodopa/benserazide		ClinicalTrials.gov Identifier: NCT03091543
	Many plants	Rutin	6-OHDA in PC-12 cells	10, 50, and 100 µM for 8 h	↑cell viability, catalase, SOD, GPx, GSH, ↓ MDA		[77]
			6-OHDA-induced PD rat model	25 mg/kg for 21 days	↑ behavior activities, GSH and dependent enzymes, DA and its metabolites, protein carbonyl, ↓ TBARS, H_2_O_2_, NO level, TNF-α and IL-1β		[78]
	Citrus trees	Hesperidin	6-OHDA-induced PD mice model	50 mg/kg for 28 days	↑ behavior activities, GSH and dependent enzymes, SOD, Catalase, DA and its metabolites ↓ROS		[79]
	*Piper nigrum*	Piperine	6-OHDA-induced PD mice model	10 mg/kg for 15 days	↑ behavior activities, GSH and dependent enzymes, SOD, Catalase, ↓ LPO, Caspase-3 and Caspase-9, TNF-α, and IL-1β		[80]

**Amyotrophic lateral sclerosis**	*Panax and Eleutherococcus*	Ginseng root	SOD1-G93A Transgenic Mouse	40, 80 mg/kg	↑ onset to clinical signs and surrogate death		[81]
		Genistein	SOD1-G93A Transgenic Mouse	16 mg/kg 2 times per day	↑ longevity, ↓ ALS symptoms, ↑ motor neurons, ↓ TLR2, TLR4, and NF-kB, p65 levels, IL-1β, IL-6, TNF-α levels		[82]
	*Curcuma longa*	Brainoil, curcumin supplement	42 randomised ALS patients	600 mg/day for 3 months	↓ disease progression, AOPPs, oxidative damage, ↑ aerobic metabolism		[83]
	*Withania somnifera*	*Withania somnifera* extract	SOD1-G93A Transgenic Mouse	5 mg of root powder p.o.	↑ longevity, motor performance, Hsp-70, Hsp-60 and Hsp-27, motor neurons, ↓ misfolded SOD1 protein, inflammation		[84]
	Green tea	EGCG	SOD1-G93A Transgenic Mouse	10 mg/kg body	↓ disease onset, ↑ survival, motor neurons, ↓ activated microglia, NF-kB and caspase-9		[85]
	*Tripterygium wilfordii and Celastrus regelii*	Celastrol	SOD1^G93A^ transfected NSC34 cells	50 nmol/L for 4 h	↑ cell viability, ↓ MDA, ↑ mRNA expressions of GCLC and GST, ERK1/2 and Akt		[86]
	Grapes and other plants	Resveratrol	BM-MSCs derived from ALS patients	1 μM for 12 h	↑ Neuro-progenitor markers, nestin, NF-M, Tuj-1, and Map-2, AMPK/SIRT1 signaling		[87]

**Huntington’s Disease**	*Panax ginseng*	Protopanaxatriol	3-NP-induced HD	5, 10, and 20 mg/kg, po	↑ weight, locomotor activity, antioxidant enzymes, HO-1, NQO1, ↓ ROS		[88]
	Many plants	melatonin	3-NP-induced HD	1 mg/kg for 8 days	↓ LPO, protein carbonyl, ↑ SOD and succinate dehyrogenase		[89]
			20 HD gene carrier subjects	5 mg/day, 30 min before bedtime/month	To improve sleep quality in HD gene carriers		ClinicalTrials.gov Identifier: NCT04421339
	Grapes and other plants	Resveratrol	SH-SY5Y cells hyper-expressing the mutant polyQ Huntingtin (polyQ-Htt) protein	100 µM for 24 h	↑ cell viability, autophagy, autophagy degradation of mutant Huntingtin, ↓ ROS		[90]
			Double blind, randomized controlled 102 early affected HD patients	800 mg/day for 1 year	To evaluate resveratrol effect on caudate volume in HD patients		ClinicalTrials.gov Identifier: NCT02336633
	Green tea	EGCG	Cell culture, yeast model and HD transgenic flies	Different doses	↓ aggregation of mutant htt exon 1 protein, polyQ-mediated htt protein aggregation, cytotoxicity		[91]
	*Vitis vinifera*	trans-(-)-ε-Viniferin	STHdhQ7/Q7, STHdhQ111/Q111, Tet-Off PC12 cells, Neuroblastoma N2a cells and Primary cortical neurons	1 nM, 10nM, 100nM, 1 µM	↓ cell toxicity, oxidative stress, mitochondrial dysfunction, ↑ mitochondrial genesis, SIRT3-dependent AMPK Activation		[92]
	*Withania Somnifera*	*W. somnifera* root extract	3-NP-induced HD	100 and 200 mg/kg for 14 days	↑ body weight, behavioral activities, SOD, catalase, mitochondrial complex (I, II, III) levels, ↓ LPO, nitrite, LDH		[93]

**Vascular Cognitive impairment**	*Aster ageratoides*	Aster ageratoides extract (AAE)	PC 12 cells treated with 50 µM glutamate or 2VO/H surgery in Sprague Dawley rats	0, 10, 25, and 50 µg/mL or 50 mg/kg b.w		↓ Memory impairment in vivo, ↓ hippocampal structures, neuronal excitotoxicity in vitro	[94]
	*Ginkgo biloba* L	bilobalide	male Sprague Dawley rats (2-vessel occlusion, 2-VO)	2, 4 and 8 mg/kg		↓ Memory impairment, ↓ nuclear condensation, ↑SOD and GSH, ↓ NOS and MDA, TNF-α	[95]
		EGb761	bilateral common carotid arteries repeated occlusion in rats and ip injection of sodium nitroprusside	50 mg/kg		↓ Memory impairment, proliferation of neural stem cells in dentate gyrus and subventricular zone	[96]
		*Ginkgo biloba* extract	90 patients, randomized, double-blind, placebo-controlled trial	60 and 120 mg		↑ CGI scores, no effect on trans cranial Doppler ultrasound, more adverse reactions in Placebo group.	[97]
	*Huperzia serrata*	Huperzine	randomized, double-blinded, placebo-controlled study with 78 patients with mild to moderate VaD	0.1-mg bid		↑ MMSE, CDR, and ADL scores	[98,99]
**Stroke (causes secondary neurodegeneration)**	Grapes and other plants	Resveratrol	Primary cortical neuron cultures with OGD/R	1, 5, and 20 µmol/L for 24 h	↑ cell viability, neurite outgrowth and synaptogenesis via Shh signaling pathway, Sirt1 activation		[100]
			(MCAO/R) Model	30 mg/kg for 7 days	↑ behavior deficits, ↓ infarction volume, ↑ NeuN+ cells, ↓ TUNEL+ cells		[101]
	*Ginkgo biloba*	Ginkgolides	NSC line	20, 40 or 60 mg/L	↑ cell viability, process length and cell body area, sizes of NSE, GFAP and SOCS2-positive cells		[102]
			mouse model of myocardial I/R injury	2.5 mL/kg	↑ cardiac function, ↓ LDH and AST, TWEAK expression, infarction volume		[102]
	*Curcuma longa*	Curcumin	Mouse N2a cells hypoxia model	5, 15, 25, and 35 μmol/L for 24 h	↑ cell viability, mitochondrial disruption, Bcl2 expression, ↓ Tunnel positive cells, Bax and Caspase-3 expression		[103]
			Rat model of global brain ischemia	25 and 50 mg/kg	↑ DA and its metabolites DOPAC and HVANE and 5-HT, cell viability, ↓ COX-2, TNF-α expression		[104]
	Green tea	EGCG	HBMVECs OGD/R model	2 *μ*M for 24 h	↑ cell viability, SOD, migration and tube formation, mRNA expression of VEGF, Bcl2, ↓ apoptosis and autophagy, ROS, LDH, MDA, mRNA expression of Bax and Caspase-3		[105]
			Rat MCAO model	20 mg/kg	↓ infarct volume, TUNEL^+^ cells, NO, MDA, ↑ Behavioral parameters, SOD and GPx		[106]
	*Scutellaria baicalensis*	Baicalin/baicalein	Rat MCAO model	200 mg/kg, 24 h after reperfusion till 7 days	↑ behavior deficits, microglia/macrophage M2 markers CD206 and CD 163, ↓infarct volume, microglia/ macrophage M1 markers CD86 and CD 16, MAPK and NF-κB		[107]
	*Angelica sinensis*	Ligustilide	Rat MCAO model	7.5, 15 or 30 mg/kg for 3 days	↑ behavior deficits, ↓ infarct volume, ↑ HSP-70 and MAPK activation		[108]
	*Sinomenium acutum*	Sinomenine	Mice MCAO model	10 or 20 mg/kg daily for 3 days	↑ behavior deficits, ↓ infarct volume, apoptosis, astrocyte and microglial activation, NLRP3 inflammasome, IL-1β, IL-6, IL-18 and TNF-α generation		[109]
	*Magnolia officinalis*	Honokiol	Rat cerebral ischemia reperfusion model	0.7–70 µg/kg, 15 min after ischemia	↓ Cerebral edema, p65 level, NO, TNF- α, RANTES/CCL5 levels		[110]
	Zingiber officinale	Zingerone	Rat MCAO model	50, 100 mg/kg at 5 h and 12 h after initiation of surgery	↑ behavior deficits, ↓ infarct volume, LPO, Caspase-3 and -9 Apaf-1, Bax, ↑ GSH and dependent enzymes, Catalase, Bcl-2		[111]
	Many plants	Perillyl alcohol	Rat MCAO model	25, 50, 100 mg/kg for 7 days	↑ behavior deficits, ↓ infarct volume, LPO, TNF- α, IL-1β, IL-6, COX-2, iNOS, NF-κB, ↑ GSH and dependent enzymes, Catalase		[112]
	*Piper nigrum*	Piperine	Rat MCAO model	10 mg/kg for 15 days	↓ infarct volume, ↑ behavior deficits and histopathological findings, ↓ TNF- α, IL-1β, IL-6, COX-2, iNOS, NF-κB		[113]

**Prion Disease**	*Scutellaria biacalenesis*	Baicalein	PrP 106-126-induced-(SH-SY5Y and PrP 106-126 and SK-N-SH) cells	80 µM	↓ROS production, ↓ mitochondrial dysfunction, ↓ apoptosis, ↓ JNK signalling		[114]
	Grapes and other plants	Resveratrol	PrP 106-126-induced-(SH-SY5Y and PrP 106-126 and SK-N-SH) cells	2–4 µM	↑ autophagy, ↓ mitochondrial dysfunction, ↓ apoptosis		[115]
	Cupressaceous plants	Hinokitiol	PrP 106-126-induced SK-N-SH	8 µM	↑ HIF-1α, ↑ autophagy, ↑ p62/SQSTM1, ↓ apoptosis		[116]
	Many plants	Rutin	PrP induced-HT22 cells	10 µg/mL	↓ ROS and NO, ↓ caspase 3 activity, ↓ caspase 8, FAS, FASL, ↑ BDNF		[117]

**Frontotemporal dementia**	Many plants	Nicotine	Grn ^-/-^mice	0.6 mg/kg P.I daily for 14 days	↓ CD68, IL-1β, CD11b, ↑ sociability		[118]
	*Curcuma longa*			250–500 µg/mL	↓ AChE		[119]
	*Piper nigrum*			250–500 µg/mL	↓ AChE		[119]
**Pick’s disease**	*Curcuma longa*	Curcumin	Primary astrocytes from NPC ^+/+^ and NPC ^-/-^ mice	30 µM	↑ cytosolic Ca^2+^, ↑ viability		[120]
	Many plants	δ-tocopherol	Human fibroblast and baby hamster kidney cells	40 µM	↓ cholesterol accumulation, ↓ lysosomal size, ↑ intracellular Ca^2+^, ↑ Ca^2+^ deficiency		[121]
	Many plants	Quercetin	Coca-2 cells and male Wistar rats	100 µM and 5 mg/kg (rats)	↓Cholesterol uptake		[122]
	Many plants	Luteolin	Coca-2 cells and male Wistar rats	100 µM and 5 mg/kg (rats)	↓Cholesterol uptake		[122]

**Spinocerebellar ataxia**		Melibiose	293 cells and SCA17 transgenic mice	100 nm - 100 µM for 6 days and daily I.P for days (24 mg/kg)	↓ polyQ aggregration. ↓ ROS, ↑ autophagy, ↓ caspase 3		[123,124]

**Spinal muscular atrophy**	*Brucea javanica*	Bruceine D	SMA mice and Δ7 mice	10 to 30 mg/kg i.p once a day for 7 days	Correcting the splice defect in SMN2, ameliorating SMN phenotype defects		[125]
	*Tripterygium wilfordii*	Triptolide	SMA mice, NSC34 and N18TG2 cells	0.1 mg/kg I.P daily	↑ SMN, Gemin2 and Gemin3 expression levels, ↑ transcription of SMN, ↑ survival rate and ↓ SMA related defects		[126]


↑prevented/loss of/induced/enhanced/improved/increased/upregulated/elicited/promoted/restored/activated. ↓down-regulated/attenuated/decreased/declined/terminated/ blocked/ prevented/ inhibited. 3xTg-AD: Enhanced susceptibility of triple transgenic Alzheimer’s disease, 5-HT: 5-hydroxytryptamine, AlCl_3_+D-gal: Aluminium chloride+d-galactose, ALP:Alkaline phosphatase, AMPA: α-amino-3-hydroxy-5-methylisoxazole-4-propionic acid, APP/PS1:amyloid precursor protein/presenilin 1, APP:Amyloid precursor protein, ARG1:Arginase, Bcl-xl: B-cell lymphoma-extra-large, CALM: Clathrin-assembly lymphoid myeloid leukaemia protein, CAMK4: Calcium/calmodulin-dependent protein kinase type IV, CAMKK1: Calcium/Calmodulin Dependent Protein Kinase Kinase 1, CREB: cAMP response element-binding protein, CXCR4: C-X-C chemokine receptor type 4, DOPAC: 3,4-Dihydroxyphenylacetic acid, GCLC: Glutamate-Cysteine Ligase Catalytic Subunit, GSH: Glutathione, GST: Glutathione S-transferase, hBMECs: Human brain microvascular endothelial cells, Hsp-27: Heat shock protein-27, Hsp-60: Heat shock protein-60, HSP-90: Heat shock protein-90, ICV-STZ: Intracerebroventricular streptozotocin, MALAT1: Metastasis Associated Lung Adenocarcinoma Transcript 1, Map-2: Microtubule associated protein 2, MCAO/R: middle cerebral artery occlusion- reperfusion, MDA: *Malondialdehyde,* MN9D: murine mesencephalon-derived dopaminergic neuronal cell line, mTORC1: mammalian target of rapamycin complex 1, MWM: Morris water maze, NE: Norepinephrine, NF-M: Neurofilament medium polypeptide, NGF: Nerve growth factor, NMDA: N-methyl-D-aspartate, NO: Nitric oxide, p-AMPK/LKB: AMP-activated protein kinase/liver kinase B, PI3K: phosphoinositide 3-kinase, PKC: Protein kinase C, RANTES/CCL5: regulated upon activation, normal T cell expressed and secreted/ Chemokine (C-C motif) ligand 5, SIRT-1: silent mating type information regulation 2 homolog 1, SNCA: Synuclein Alpha, TBARS: Thiobarbituric acid reactive substances, TH^+^: Tyrosine hydroxylase, TLR2: Toll-like receptor 2, TLR4: Toll-like receptor 4, TrkB: Tropomyosin receptor kinase B, Tuj-1: Neuron-specific Class III β-tubulin, TUNEL^+^: terminal deoxynucleotidyl transferase-mediated dUTP nick-end labelling^+^, TWEAK: TNF-related weak inducer of apoptosis, γ-GT: Gamma-glutamyl transferase.
